# *Notes from the Field:* Increase in Drug Overdose Deaths Among Hispanic or Latino Persons — Nevada, 2019–2020

**DOI:** 10.15585/mmwr.mm7119a4

**Published:** 2022-05-13

**Authors:** Shawn A. Thomas, Amanda T. Dinwiddie, Elyse Monroy

**Affiliations:** ^1^University of Nevada, Reno, School of Public Health, Reno, Nevada; ^2^Division of Overdose Prevention, National Center for Injury Prevention and Control, CDC.

Reports have documented national and state-level increases in drug overdose–related emergency department visits, emergency medical services incidents, and deaths among racial and ethnic minority groups in the United States during 2020 amid the COVID-19 pandemic (*1*–*3*). In June 2021, the Nevada Department of Health and Human Services reported an increase in drug overdose deaths during 2020 among Hispanic or Latino (Hispanic) persons, who make up approximately one third of Nevada’s population (*4*). To better understand this increase, investigators analyzed 2019–2020 State Unintentional Drug Overdose Reporting System (SUDORS) data.*

SUDORS is a CDC surveillance system containing detailed information on death scene investigations, toxicology, and other risk factors associated with fatal drug overdoses of unintentional or undetermined intent. Hispanic persons were identified through the SUDORS ethnicity variable, which considers persons with Mexican, Puerto Rican, Cuban, Central or South American, or other Spanish culture or origin as Hispanic, regardless of race. Chi-square pairwise comparisons with Bonferroni adjustment were used to assess differences between 2019 and 2020 in characteristics and circumstances for all overdose deaths and for those among Hispanic persons (*5*).

From 2019 to 2020, drug overdose deaths among all races and ethnicities in Nevada increased 54.5% (from 510 to 788), compared with 119.7% (from 66 to 145) among Hispanic persons (Table). By sex and age group, the highest percentage increases occurred among males (overall = 6.9%; Hispanic persons = 6.2%) and those aged <25 years (overall = 77.6%; Hispanic persons = 86.2%). During 2020, the proportions of Hispanic decedents who were male (77.2%) and those aged <25 years (28.3%) were higher than overall proportions (68.3% male; 13.5% aged <25 years). From 2019 to 2020, the proportion of deaths involving illicitly manufactured fentanyls^†^ increased significantly overall (115.6%) and among Hispanic persons (134.5%). By route of drug administration, oral ingestion was highest for deaths in both 2019 (overall = 38.0%; Hispanic persons = 40.9%) and 2020 (overall = 40.7%; Hispanic persons = 31.0%), and the proportion of smoking among Hispanic persons increased 31.6%. During 2020, among those with opioids contributing to death (opioid-involved deaths), only 28.1% of all decedents and 35.7% of Hispanic decedents were known to have received naloxone (Table). Data in SUDORS are dependent on information documented at time of death, and some might be missing, which could underestimate some of the percentages reported. In addition, Hispanic deaths are included in the “all deaths” group.

**TABLE:** Characteristics, circumstances, and substances contributing to drug overdose deaths among all decedents and Hispanic or Latino decedents — Nevada, 2019–2020

Characteristic, circumstance, or substance	All deaths			Hispanic or Latino deaths		
	2019 (n = 510) No. (%)	2020 (n = 788) No. (%)	% Change in proportion from 2019 to 2020	2019 (n = 66) No. (%)	2020 (n = 145) No. (%)	% Change in proportion from 2019 to 2020
**Proportion of overdose deaths among Hispanic or Latino persons, %**	NA	NA	NA	12.9	18.4	42.6
**Sex**						
Male	326 (63.9)	538 (68.3)	6.9	48 (72.7)	112 (77.2)	6.2
Female	184 (36.1)	250 (31.7)	−12.2	18 (27.3)	33 (22.8)	−16.5
**Age group, yrs**						
<25	39 (7.6)	106 (13.5)	77.6^*^	10 (15.2)	41 (28.3)	86.2
25–34	83 (16.3)	149 (18.9)	16.0	15 (22.7)	42 (29.0)	27.8
35–44	99 (19.4)	144 (18.3)	−5.7	12 (18.2)	27 (18.6)	2.2
45–54	120 (23.5)	158 (20.1)	−14.5	18 (27.3)	16 (11.0)	−59.7
≥55	169 (33.1)	231 (29.3)	−11.5	11 (16.7)	19 (13.1)	−21.6
**Location of overdose^†^**						
Home	391 (76.7)	604 (76.6)	−0.1	55 (83.3)	108 (74.5)	−10.6
Other location or unknown	119 (23.3)	184 (23.4)	0.4	11 (16.7)	37 (25.5)	52.7
**Evidence of unresolved substance use or misuse problem reported^¶^**						
Yes	350 (68.6)	536 (68.0)	−0.9	46 (69.7)	91 (62.8)	−9.9
No or unknown	160 (31.4)	252 (32.0)	1.9	20 (30.3)	54 (37.2)	22.8
Route of administration**						
Oral ingestion	194 (38.0)	321 (40.7)	7.1	27 (40.9)	45 (31.0)	−24.2*
Injection	109 (21.4)	126 (16.0)	−25.2*	11 (16.7)	16 (11.0)	−34.1
Smoking	92 (18.0)	147 (18.7)	3.9	10 (15.2)	29 (20.0)	31.6
Snorting	26 (5.1)	82 (10.4)	103.9*	—**^§^**	19 (13.1)	—**^§^**
Unknown	107 (21.0)	51 (6.5)	−69.0*	17 (25.8)	19 (13.1)	−49.2*
**Opioids contributing to death††**						
Any opioid	293 (57.5)	516 (65.5)	13.9*	37 (56.1)	98 (67.6)	20.5
Prescription opioids	128 (25.1)	180 (22.8)	−9.2	18 (27.3)	23 (15.9)	−41.8
Heroin	103 (20.2)	124 (15.7)	−22.3	—**^§^**	18 (12.4)	—**^§^**
Illicitly manufactured fentanyls**^§§^**	75 (14.7)	250 (31.7)	115.6*	13 (19.7)	67 (46.2)	134.5*
**Naloxone administration among decedents for whom opioids was listed as a cause of death**						
Yes	73 (24.9)	145 (28.1)	12.9	12 (32.4)	35 (35.7)	10.2
No or unknown	220 (75.1)	371 (71.9)	−4.3	25 (67.6)	63 (64.3)	−4.9
**Nonopioids contributing to death^††^**						
Methamphetamine	264 (51.8)	376 (47.7)	−7.9	35 (53.0)	67 (46.2)	−12.8
Benzodiazepines	89 (17.5)	168 (21.3)	21.7	—**^§^**	35 (24.1)	—**^§^**
Cocaine	53 (10.4)	87 (11.0)	5.8	—**^§^**	19 (13.1)	—**^§^**

**Source:** Nevada State Unintentional Drug Overdose Reporting System 2019–2020.

**Abbreviation:** NA = not applicable.

^* ^Indicates statistically significant change from previous year (p<0.01).

^†^ House, home, or apartment setting might not be the decedent’s residence. Other locations include motor vehicle, park, or other outdoor area that is not in a home.

^§^ Indicates count or percentage change suppressed because of sample size <10.

**^¶^** Evidence of unresolved substance use or misuse problem reported does not include alcohol.

** Based on information from death scene, witness, and autopsy evidence that describe how the fatal overdose substance(s) could have been administered (e.g., pills found at the scene to indicate oral ingestion), which might or might not include substances documented as contributing to death; categories are not mutually exclusive.

^††^ Includes substances listed as a cause of death. Multiple substances could be listed as a cause of death; therefore, substances are not mutually exclusive.

^§§^ Illicitly manufactured fentanyls include both illicitly manufactured fentanyl and illicit fentanyl analogs; they were identified using both toxicology and scene evidence because toxicology alone cannot distinguish between them.

To improve evidence-based drug overdose prevention and response efforts among persons who use opioids in Nevada, particularly among young male Hispanic persons who have experienced overdoses involving illicitly manufactured fentanyls, a better understanding of the underlying risks for the recent increase in overdose deaths is needed. Naloxone can reverse the effects of overdose from opioids, such as illicitly manufactured fentanyls. Although evidence of naloxone administration for opioid-involved deaths was higher among Hispanic persons in 2020, only approximately one in three of those Hispanic decedents had evidence^§^ of naloxone administration. Test strips for detecting fentanyl might be useful, especially in substances that might be ingested or smoked. Increasing awareness of the life-saving potential of naloxone and fentanyl test strips and ensuring that persons who use drugs and their friends and family have access to both and carry them could prevent some overdose deaths. With CDC assistance, part of the Nevada Overdose Data to Action (NV OD2A)^¶^ program’s role is to provide partner organizations with data to alert corresponding communities about drug overdose death increases. For example, NV OD2A convenes community leaders to expand harm reduction strategies among younger Hispanic persons and increase naloxone access to help prevent future overdose deaths. NV OD2A also evaluates existing overdose prevention messaging in the Hispanic community to make improvements, and future actions will include continued monitoring of drug overdose deaths and working toward implementing enhanced prevention efforts.

## Footnotes

* SUDORS is a CDC surveillance system funded through Overdose Data to Action (OD2A) that supports 48 jurisdictions to collect and abstract data on unintentional and undetermined intent drug overdose deaths from death certificates and medical examiner or coroner reports (e.g., scene findings, autopsy reports, and full postmortem toxicology findings) for entry into a web-based CDC platform that is shared with the National Violent Death Reporting System (NVDRS). https://www.cdc.gov/drugoverdose/foa/state-opioid-mm.html

† Illicitly manufactured fentanyls include both illicitly manufactured fentanyl and illicit fentanyl analogs; they were identified using both toxicology and scene evidence because toxicology alone cannot distinguish between them.

§ Evidence of naloxone administration includes documentation that the decedent was administered naloxone in response to their fatal overdose.

¶
https://nvopioidresponse.org/od2a/
